# Broad‐Spectrum Diffusion Coefficient Measurements via Surface Plasmon Resonance: From Thermodynamics to Protein Conformational Disorders

**DOI:** 10.1002/cphc.202500138

**Published:** 2025-06-29

**Authors:** Giuseppe Stefano Basile, Damiano Calcagno, Nunzio Tuccitto, Diego Sbardella, Giuseppe Grasso

**Affiliations:** ^1^ Department of Chemical Sciences University of Catania Viale Andrea Doria 6 95125 Catania Italy; ^2^ Scuola Superiore di Catania University of Catania Via Valdisavoia 9 95123 Catania Italy; ^3^ IRCCS‐Fondazione Bietti Via Livenza 3 00198 Rome Italy

**Keywords:** aggregation, conformational disorders, continuum mechanics, diffusion, surface plasmon resonance, Taylor analysis, thermodynamics

## Abstract

Information regarding the dimension and the shape of molecules in solution represents a holy grail for chemists. Recently, newly designed surface plasmon resonance (SPR) methods to precisely measure the diffusion coefficients (*D*) (D‐SPR) have been developed and applied successfully to a variety of molecules, ranging from diverse long‐chain alcohols to protein conformers and oligomers involved in conformational disorders, mostly represented by neurodegenerative processes of nervous tissues of the brain and retina. The dependence of *D* on the molecular size, shape, and oligomerization state of different molecules has been widely investigated, opening up new avenues and tools for chemical, biochemical, and clinical research. Herein, the historical basis and the development of the newly proposed D‐SPR method are briefly described to obtain meaningful information about molecular features that are otherwise hard to characterize using more common and traditional bioanalytical approaches.

## Graphical abstract

Recent advancements in surface plasmon resonance protocols and simulations have enhanced the measurement and prediction of diffusion coefficients (D) for chemical species in microfluidic channels. These developments enable precise determination of D values for molecules ranging from a few angstroms to nanometric aggregates, facilitating detailed insights into molecular and aggregation properties.

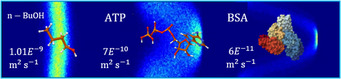

## 1 Introduction

Thermodynamics insights into material and life science phenomena drive an enormous amount of applied and fundamental research projects. It is known that whenever there is more than one chemical component in a system, the evolution of the equilibrium or nonequilibrium conditions of such a system finds its driving force in the difference between the chemical potential of each component in different regions of space.^[^ ^1^, ^2^, ^3^ ^]^

There are countless examples to support this statement both in materials chemistry, where the difference in chemical potentials between phases drives the evolution of concentration profiles of different ions,^[^ ^4^ ^]^ and in life sciences, where the diffusion of chemical species in confined or isotropic spatial conditions guides signaling patterns, metabolic pathways^[^ ^5^ ^]^ and, in some cases, determines if a cell will die or remain viable.^[^ ^6^, ^7^ ^]^

Some examples even extend to highly interdisciplinary areas of science and engineering like information theory, as proven by the rising interest in molecular communication among chemists and engineers.^[^ ^8^ ^]^

In all these cases, there is a common denominator that guides the interpretation and design of new experiments, and it is the idea that molecules flow from one region of space to another in order to minimize the total free energy of the system under the appropriate conditions. This process, when referring to the description of mass flow and concentration profiles evolution, is commonly known as diffusion.^[^ ^2^ ^]^

It is a popular misconception that concentration gradients drive such molecular flows, as this is the case only if the diffusing molecules are perfectly miscible and diluted in a solvent, as opposed to the more general case where diffusion is guided by chemical potential gradients.^[^ ^1^ ^]^

In diluted conditions, however, we find that the flow of molecules per unit surface (the number of molecules that traverse a surface per unit time) is proportional to the concentration gradient through a physical parameter named diffusion coefficient (*D*).

Diffusion coefficients are connected to almost any description of mass‐transport phenomena, and they play a pivotal role in determining the behavior of multicomponent systems. Analytical measurement and physical interpretation of *D* values are fundamental to correctly describe the diffusive properties of reacting systems, biological structures, and nanomaterial devices.

Over the course of the last 75 years, several increasingly sophisticated measurement methods have been developed, allowing for novel correlations between *D* and other physical quantities to be predicted and observed.^[^ ^9^ ^]^ This work aims to be a primer for the reader on the historical developments and current state of the art for *D* values determination via real‐time, non‐tracing and fluid‐dynamics based approaches through surface plasmon resonance (SPR) instruments In the following, after a concise treatment of the basic methods, further developments in this branch of chemistry and possible implications for future avenues of research will be presented.

## 2 Taylor–Aris Dispersion Analysis

As a general reminder, from now on, we will talk about isotropic diffusion, meaning that we assume *D* to be a scalar quantity, and molecules do not have a preferred flowing direction in the absence of external fields (like electromagnetic fields or pressure gradients).

Given these premises, it is possible to say that there are almost more methods to measure *D* values than researchers willing to count them, but not all of them are equivalent, meaning that, although most authors might claim to be measuring *D*, it is to be noted that without explicitly reporting the experimental technique employed and the experimental conditions, numerical results for the same types of molecules can be drastically different, as *D* is generally a function of temperature, concentration and number of different diffusing species.

Bearing that in mind, we might distinguish two different approaches to measure diffusion coefficients: direct and indirect ones.

The main difference between these two is that the former makes use of Fick's first law^[^ ^2^, ^10^ ^]^

(1) $$\begin{array}{c} J\left(x\right)=-D\nabla C\left(r\right) \end{array}$$

where *x* = (*x*, *y*, *z*) represents the spatial coordinate and *t* is the time, *J*(*x*) is the flow of molecules across a surface of unit area, and ∇*C* is the concentration gradient.

It is difficult to measure concentration gradients and matter flows directly in diluted solutions (the only ones for which Fick's first law does hold as it is), and for this reason a great number of methods uses instead the indirect approach, through the application of the more general Fick second law, that relates *D* to the time evolution of the concentration profile, or of one of its generalizations.

(2) $$\begin{array}{c} \frac{\partial C\left(x,t\right)}{\partial t}=D\nabla^{2}C\left(x,t\right) \end{array}$$

Some methods, on the other hand, rely on the measurement of other properties that are related to *D* on a microscopic level.

There are several ways on multiple‐dimensional scales to interpret the physical significance of *D*, and, for any macroscopic theory based on continuum mechanics arguments, there is a corresponding microscopic theory that relies on the statistical properties of the large number of molecules diffusing in a sample. Molecules at finite temperature are subject to a constant Brownian stochastic motion, and we can define quantities such as their mean square displacement or their velocity autocorrelation function.^[^ ^11^ ^]^

Both of these quantities are of direct fundamental theoretical interest for *D* prediction in molecular dynamics or Monte–Carlo simulations. Furthermore, the abovementioned quantities find indirect application in experiments such as diffusion ordered nuclear magnetic resonance (NMR) Diffusion‐ordered spectroscopy (DOSY) and dynamic light scattering (DLS)^[^ ^12^, ^13^, ^14^ ^]^ to determine *D* values.

A drawback of these techniques is that they usually require either marked species and/or a really careful setup of the samples to avoid convection due to thermal gradients, albeit being among the most useful historical methods to determine *D*.

In the following, we will focus on another way to obtain *D* through pure hydrodynamics considerations that, although requiring the absence of strong temperature gradients, needs only that the samples are completely free from dust particles or bubbles.

Before addressing the new SPR‐based approach, it is best to introduce the original theory of Taylor dispersion analysis (TDA).^[^ ^15^ ^]^

To address this method in the best way possible, it is mandatory to consider some preliminary theoretical facts.

The second Fick law is derived in any good textbook dealing with the mechanics of continuum bodies, such as McQuarrie's, and a full mathematical treatment of this kind of equation is found in Crank's monography.^[^ ^2^, ^16^ ^]^

Let us suppose now to have a cylindrical system where we can apply a pressure gradient to allow a certain velocity field ***u*** (***x***, *t*). In these conditions, it is provable that the governing equation (from now on, advection–diffusion equation) for a single diluted component is

(3) $$\begin{array}{c} \frac{\partial C\left(x,t\right)}{\partial t}=-\nabla \cdot J\left(x,t\right)=D\nabla \cdot \left(\nabla C\right)-\nabla \cdot \left(Cu\right) \end{array}$$

where ***J*** is the matter flow across a unit area surface, composed of the two additive contributions of the velocity field and the diffusive field. It is impossible to overemphasize the fact that this equation is essentially wrong as long as we step into the regime of highly interacting multiple solutes, and if we consider solutions so concentrated that the velocity field starts to couple with the different diffusive flows. A better theory that reduces to Equation (3) in the case of one single diluted solute is that of linear nonequilibrium thermodynamics, for example, in the formalism of Onsager and as developed by many authors over the last century,^[^ ^1^, ^17^, ^18^ ^]^ but such treatment would be out of scope for this work.

Let us assume that Equation (3) holds instead, and that the velocity field is only oriented along the *z* direction (i.e., the axial direction of the cylinder). Furthermore, let us consider that the flow is incompressible and follows the parabolic velocity field given by the Hagen–Poiseuille law. In these conditions, Equation (3) reduces to

(4) $$\begin{array}{c} \frac{\partial C\left(x,t\right)}{\partial t}=D\nabla_{r,\theta ,z}^{2}C-u{\left(r\right)}_{z}\cdot \nabla_{z}C \end{array}$$

(5) $$\begin{array}{c} u\left(r\right)=u_{\text{max}}\left(1-\frac{r^{2}}{R}\right)e_{z} \end{array}$$

where we shifted to cylindrical coordinates so that now *x* = (*x*, *θ*, *z*) and the velocity field ***u*** (*r*) is only along the Z direction and depends parabolically on *r*. This simplifies greatly the treatment of diffusion–advection equations in cylindrical channels, and a general analytical solution to this problem, assuming arbitrary release of solute concentration profiles at *t* = *t*_0_ has been developed by Schafer et al. in the context of molecular communication.^[^ ^19^ ^]^

In 1953, Taylor proposed a capillary technique to measure diffusion coefficients with unprecedented accuracy for the period, developing its theory by considering the possibility of sending a pulse of sample, ideally represented by a Dirac distribution at time zero, from a source to a detector in a microcapillary tube.

Taylor's ideas were based on the concept that there is a certain interval of hydrodynamic parameters determined by the liquid flow rate and the length of the cylinder, such that the only relevant effect for diffusive phenomena would be in the radial direction and decoupled from the advection happening in the Z direction.

In this regime, given a suitable experimental setup, comprising a detector able to transduce a signal proportional to the concentration profile, Taylor found that the concentration profile in cylindrical channels under the Hagen–Poiseuille velocity profile and considering a plug of initial concentration ***C*** (*x*, *t* = 0), would have either the form of a modified error function or modified Gaussian, due to the relation of the solution of Equation (3) with the normal distribution under the appropriate boundary conditions.

**Figure 1:**
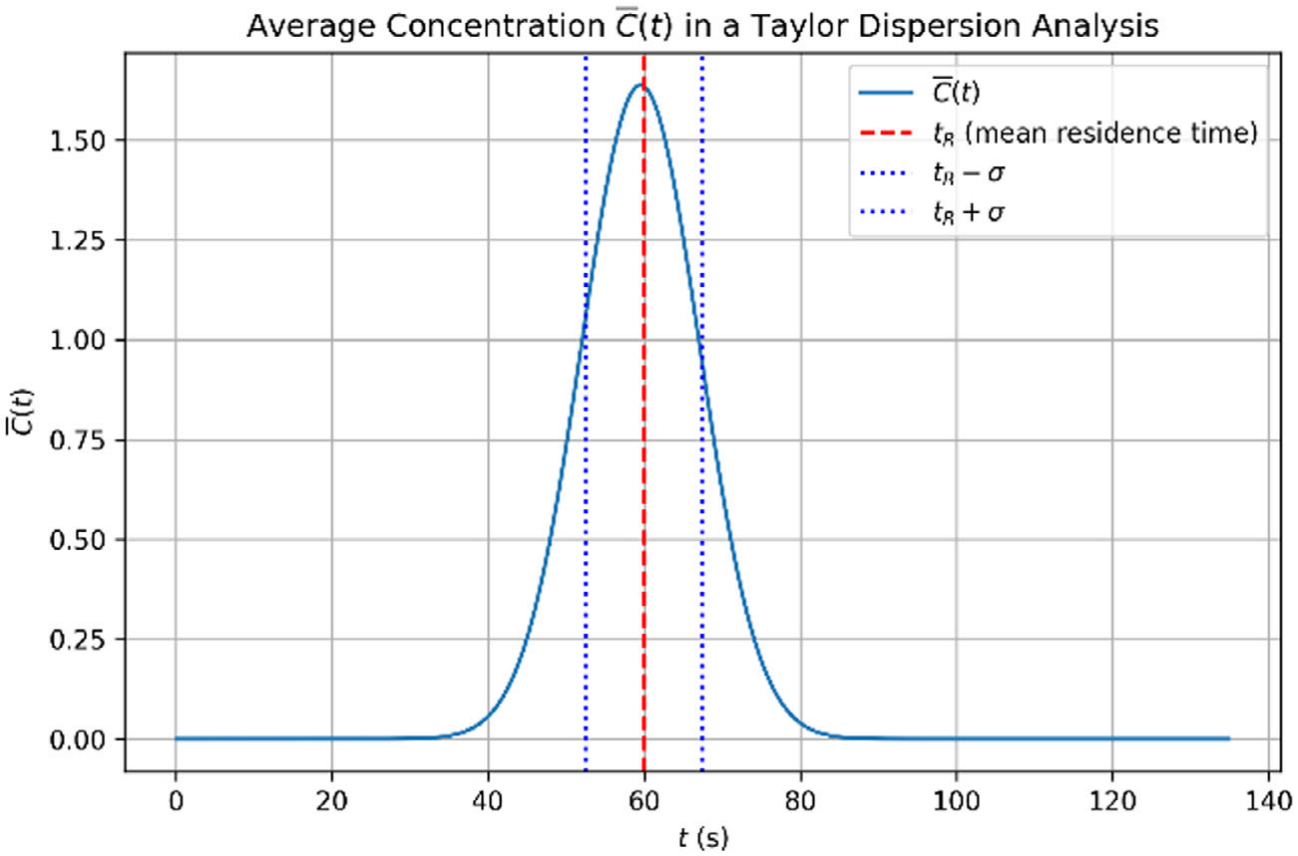
Theoretical time evolution of the average solute concentration at the detector following the injection of an ideal Taylor–Aris pulse. The red dash‐dotted line indicates the mean residence time, while the blue dotted lines mark the inflection points of the approximately Gaussian profile.

Under the assumptions of Taylor and as explained by Aris^[^ ^20^ ^]^ in a later, more comprehensive, treatment, one can relate the signal transduced from a detector to the concentration profile evolution over time, finding that, given a long enough tube and meeting the necessary conditions on the hydrodynamic parameters required to have Taylor diffusion, the signal evolves as^[^ ^21^ ^]^

(6) $$\begin{array}{c} S\left(t\right)\propto \bar{C}\left(t\right)\approx \frac{M}{2\pi^{3/2}R^{2}}\frac{1}{\sqrt{kt}}e^{-\frac{{\left(t-t_{r}\right)}^{2}}{\sigma^{2}}} \end{array}$$

where $\bar{C}\left(t\right)$ is the average concentration profile along a circular cross section of a channel, *M* is the total mass of analyte inside the section, *t*_r_ is the mean retention time of the analyte, *σ* ^2^ is the temporal variance of the concentration time evolution profile, and *k* is a geometrical factor. A graphical representation of Equation (6) is reported in **Figure** **1**.

Under these conditions, *D* can be computed as

(7) $$\begin{array}{c} D=\frac{t_{r}^{2}}{24R^{2}\sigma^{2}} \end{array}$$

which holds only when the length of the tube is sufficiently large to avoid the contribution from advection in the z‐axis to prevail over the radial diffusion.

## 3 Diffusion coefficients‐SPR (D‐SPR) Analysis: Going Beyond Classical Taylor‐Aris Dispersion through SPR Approaches

SPR is a commonly employed optical‐hydrodynamics‐based technique in analytical and biophysical chemistry. Its main advantages over most other instrumental methods reside in the label‐free treatment of analytes, reduced sample volumes, and the ability to be coupled with complex, finely tunable chemistries in the sensor chamber to allow for finely tuned kinetics and thermodynamics analyses of biological interactions.^[^ ^22^, ^23^, ^24^, ^25^ ^]^

SPR systems for biophysical and biochemical analytical procedures have usually the following setup: an injection system based on some kind of peristatic pump coupled with a loop system and *n*‐way valves able to accurately and effectively measure and inject fixed volumes of sample and ensure a continuous flow of buffer, a microfluidic system, possibly with multiple channels, that connects the injection block to the flow cell, where a sensor meeting the physical and geometrical criteria for SPR signal generation is placed.^[^ ^26^, ^27^ ^]^

The typical output of an SPR experiment is the sensorgram: a graph showing the total internal reflection angle evolution over time. This signal is proportional to the refractive index change of the sample flowing over the sensor in the flow cell, which in turn is proportional to the concentration profile and to the interactions happening at the interface between the sensor and the chemical species in solution.

In standard SPR experiments, there is a constant need for the removal of mass transfer contributions to the signal, which would compete with the kinetics and alter the rate constant evaluations.

To leverage the contribution of mass transfer as a means to get new insights into biological phenomena, various experimental protocols have been designed to carry out diffusion experiments both in the Taylor–Aris conditions^[^ ^28^ ^]^ and not.^[^ ^29^, ^30^, ^31^ ^]^

Considering a constant cross‐sectional microfluidic channel long enough to allow for radial solute diffusion to be appreciable, various methods have been derived to infer *D* values from the sensorgrams.

Although specific ad‐hoc experimental setups are documented in the literature, like in Loureiro et al.^[^ ^28^ ^]^ there is no simple way to exploit Taylor–Aris dispersion in a conventional SPR experiment. Thus, a little shift in paradigm is necessary to adapt the experiment for a wider range of users.

In typical SPR setups, the microfluidic system is not designed to release short pulses of analyte; thus, it is not usually possible to use Equation (6) to derive *D* values due to the different boundary conditions on the diffusion–advection equation.

It is, however, quite straightforward to set up an injection profile that releases a plug of analyte with a precise volume through the usage of loop systems and multiple n‐way valves.

**Figure 2:**
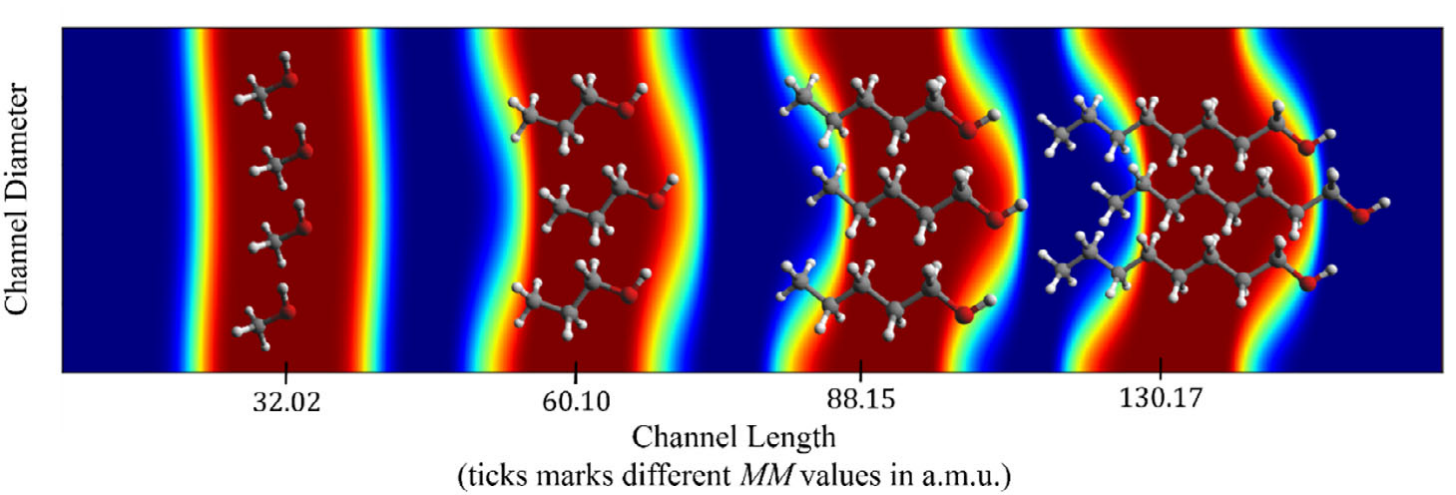
Schematic illustration of alcohol diffusion within the tubing system during a D‐SPR experiment. The x‐axis ticks represent MM (a.m.u.) of the solute, while analyte plugs are color coded by concentration, with red indicating the highest concentration and blue representing zero. The vanishing wave is omitted for clarity. Simulations were performed using finite difference computational schemes with a time step of 8000 ms over 100 steps. The only variation among simulations is the diffusion coefficient (*D*) used in the governing equation. Reproduced with permission.^[^ ^9^ ^]^

Such an initial concentration profile is equivalent to a constant release of a homogeneous concentration of analyte over time, or, in other words, it is a square concentration profile released at *t* = 0 that evolves through diffusion–advection over time before reaching the SPR flow cell. A depiction of this phenomenon can be seen in **Figure** **2**.

It has been proven that, under ideal initial conditions of a square sample plug and assuming that the analyte does not interact with the sensor surface, the concentration profile integrated in the volume sampled by the plasmon evanescent wave is proportional to the signal. The proportionality between those two quantities is, however, quite tricky to formalize, as there are no accepted universal models that describe the refractive index of a multicomponent solution in nonideal systems.^[^ ^32^ ^]^

## 4 Original D‐SPR Setup

Starting from the aforementioned assumptions and using a common benchtop SPR instrument, Zingale et al. proposed a new experimental protocol, named D‐SPR, able to determine *D* values with remarkable accuracy, without the need for specialized instruments.^[^ ^29^ ^]^

**Figure 3:**
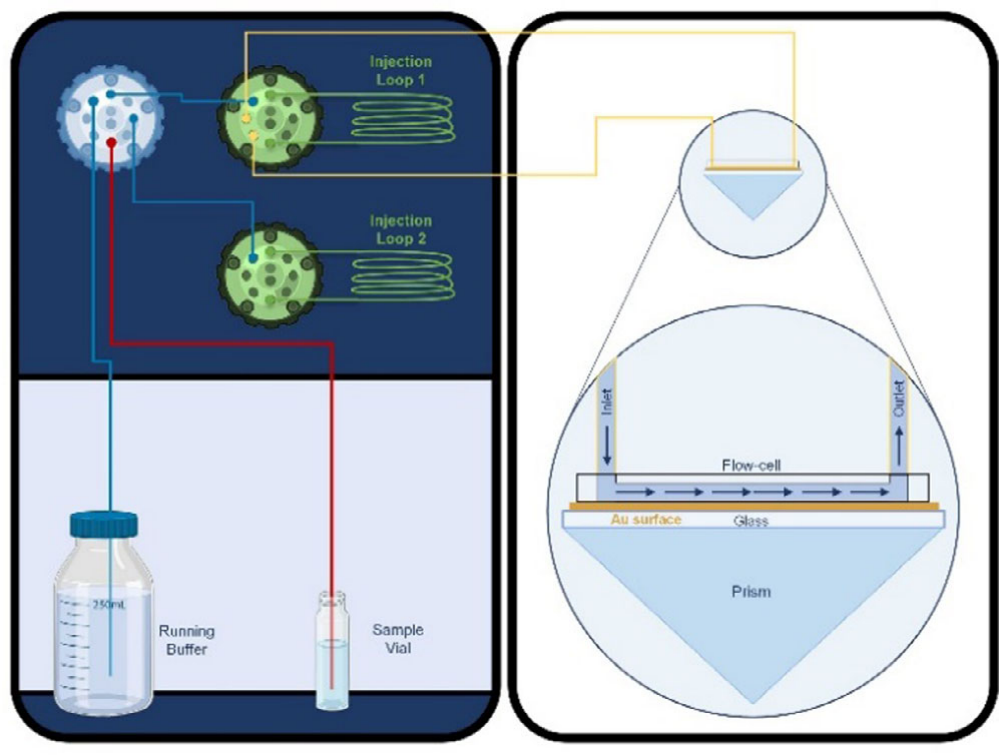
Schematic representation of a typical SPR apparatus for both standard and D‐SPR analyses. Reproduced with permission.^[^ ^31^ ^]^ In the right inset, a schematic SPR flow cell is depicted along with its associated gold sensor. The laser rays are omitted for clarity.

A schematic depiction of a typical SPR apparatus for D‐SPR measurements is reproduced in **Figure** **3**. Experimentally, the approach is quite straightforward: once the working concentration, an appropriate injection volume profile, and a suitable sensor have been selected—determined by factors such as sample availability, the chemical nature of the involved species, and their interaction with the sensor surface—a series of repeated sample injections must be performed at a flow rate on the order of 5–10 μL min^−1^ while collecting the associated sensorgrams.^[^ ^29^ ^]^

In selecting an appropriate sensor for a given analyte, the surface chemistry must be finely tuned to preclude nonspecific adsorption or unintended interactions. This criterion is typically assessed by verifying that the baseline signal reverts to its premeasurement level and by scrutinizing the morphology of the response curves prior to plateau onset. Unmodified (bare) gold sensors are generally appropriate for low‐molecular‐weight analytes such as ethanol or adenosine triphosphate, whereas surface functionalization is often requisite for the reliable sensing of proteins, peptides, or other larger biomolecules.

Several additional steps must be taken to ensure reproducibility, including system conditioning through pre‐injections and verifying that no unintended interactions occur with the sensor surface. These aspects are described in greater detail in a recent protocol by Zingale which also covers sensors and sample preparation.

**Figure 4:**
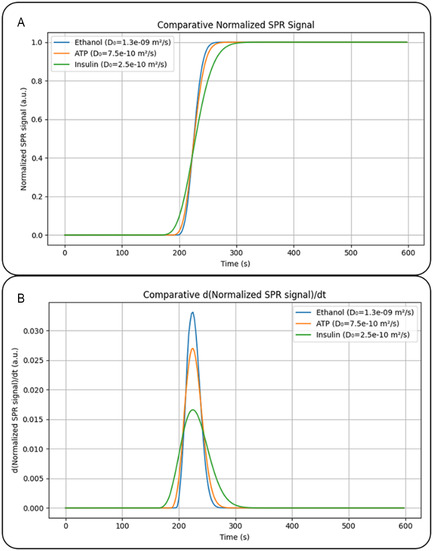
A) Simulated SPR curves and B) their corresponding first derivative signals for Ethanol, ATP, and Insulin that are shown as lines in blue, orange, and green, respectively.

Data analysis and interpretation are fundamentally based on the concepts introduced by Taylor and Aris. However, instead of considering a concentration pulse at time *t* = 0, the D‐SPR variation assumes that the average concentration in the sampling volume of the flow cell evolves according to a perturbed error function, as shown in panel A of **Figure** **4**. This approach implies that the first derivative (Panel B of Figure 4) of the SPR signal can be fitted to an exponentially modified Gaussian function

(8) $$\begin{array}{c} \frac{d}{dt}S\left(t\right)=\frac{1}{\tau }e^{-\frac{t-\mu }{\tau }+\frac{\sigma }{\tau }}\Phi \left(\frac{t-\mu }{\sigma }-\frac{\sigma }{\tau }\right) \end{array}$$

Where Φ is the standard error function, while *τ*, *μ* *σ* are fitting parameters that allow us to compute the mean (*m*), the variance (*S*), and the skewness ($\bar{sk}$) of the fitted derivative.

From these, *D* is computed as

(9) $$D_{\text{SPR}}=\frac{R^{2}m}{24s^{2}}\left(1+\bar{sk}\right)$$

All the derivations and mathematical considerations on these results are better described in the original publication and in subsequent ones.^[^ ^9^, ^31^ ^]^ Since its appearance in 2023, this approach has been further refined and employed for a number of relevant applications ranging from batteries^[^ ^33^ ^]^ to protein conformational dynamics and aggregation.^[^ ^30^, ^34^ ^]^

## 5 Recent Developments, Applications, and Variants of D‐SPR

D‐SPR has been shown to work remarkably well for small solutes in aqueous solutions, allowing the users to model the diffusive properties of homologous classes of molecules as a function of their physical properties, as shown by us in ref. [9].

In the aforementioned work, we developed a coupled simulation and experimental workflow to correlate the diffusive properties of organic linear chain alcohols in water to various extensive molecular properties such as molecular weight (MM) and configurational entropy.

Starting from Eyring's theory of absolute rate processes,^[^ ^35^, ^36^ ^]^ we were able to derive and test a fitting model for *D* (*x*) with *x* representing any molecular property linearly scaling with MM for homologous classes of linear molecules in water.

**Figure 5:**
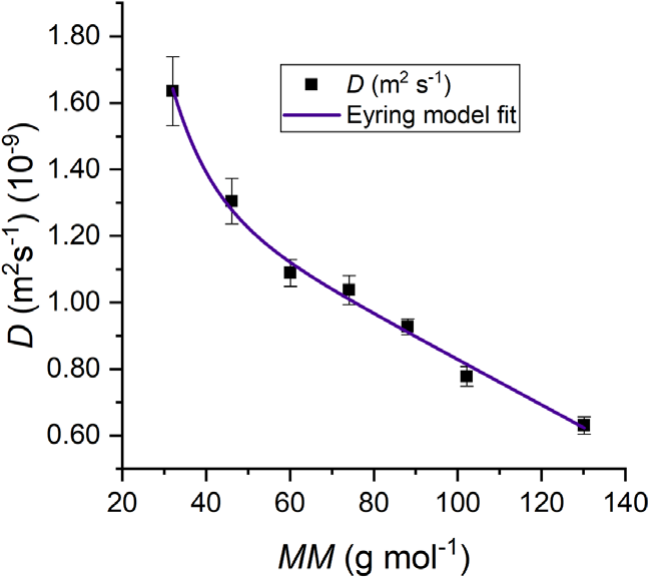
Diffusion coefficient (*D*) versus MM for linear‐chain alcohols in water, determined by D‐SPR. The violet curve represents the non‐linear fit based on an Eyring‐based phenomenological model. Data and fitting details Reproduced with permission,^[^ ^9^ ^]^ which also contains a comprehensive survey of prior models and fitting methodologies.

This “Eyring‐based” model has shown remarkable accuracy in describing a number of data trends for linear chain molecules in water (as reported in **Figure** **5**) and organic solvents,^[^ ^9^ ^]^ and a first test of its reliability was feasible thanks to the accuracy of the D‐SPR protocol.

Furthermore, guided by the simulations, we devised a data‐analysis protocol to plot *D* as a function of geometrical descriptors such as gyration radii and eccentricity, showing how the simple Stokes–Einstein equation^[^ ^37^, ^38^ ^]^ could easily fail in describing the subtle behavior of *D* for small solutes in water.

It is noteworthy that a rapid, accurate, and label‐free method for determining diffusion coefficients is available, given the strong interplay between diffusivity and the conformational properties of molecules. The ability to measure these coefficients with ease greatly simplifies the testing of theoretical models, which in turn is essential for rationalizing and modeling physicochemical trends, such as the average impact of intermolecular interactions between solutes and solvent on the solute's diffusive behavior.

In particular, we have demonstrated that one of the fitting parameters, *R*, can be correlated with the chemical nature of both the solvent and the solutes, as described by Basile et al.^[^ ^9^ ^]^ and briefly summarized in **Table** **1**. More recent advances in the D‐SPR protocol were directed in applying it to larger and more complex biological systems, whose *D* values range in the order of $1\times 10^{-11}\text{ to}\;1\times 10^{-10}{\text{ m}}^{2}{\text{ s}}^{-1}$. With these parameters and with highly diluted solutions, the concentration profile evolution over time is such that the sampling depth of common SPR systems is not able to transduce a signal that captures the full diffusion process, meaning that a simple fitting of the signal's first derivative would not be enough to accurately derive *D*.

**Table 1:** Resuming table summarizing the R fitting parameters for homologous series of molecules in various solvents under different conditions, showing how D‐SPR enables systematic and straightforward investigations of analogous chemical systems. Starting from the second row, the table compares D‐SPR results with literature reference values.

$D_{\mathrm{solvent}}^{\mathrm{solute}}$	$D_{H_{2}0}^{n-\mathrm{ROH}}$	$D_{H_{2}0}^{n\mathrm{-RCO}O^{-}}$	$D_{C_{6}H_{12}}^{n-\mathrm{ROH}}$
*R* a)	0.34 ± 0.06	0.25 ± 0.03	0.15 ± 0.01
**Solute**	**Ethanol**	**Insulin (pH 7.3)**	**ATP**
**Units**	10^−9^ m^2^ s^−1^	10^−9^ m^2^ s^−1^	10^−9^ m^2^ s^−1^
***D***_**D–SPR**_	1.30 ± 0.07a) 1.02 ± 0.02b)	0.16 ± 0.02b)	0.75 ± 0.03b)
***D***_**literature**_	≈1.05–1.24c)	0.16 ± 0.10c)	≈0.76c)

a) Data retrieved from Basile et al.^[^ ^9^ ^]^

b) Data retrieved from Zingale et al.^[^ ^31^ ^]^

c) Data retrieved from Landolt–Börnstein,^[^ ^45^ ^]^ Jensen et al.^[^ ^46^ ^]^ Bowen et al.^[^ ^47^ ^]^

To address the inherent challenges in sampling and evaluating the *D* of biological macromolecules under physiological conditions, several techniques have been used over the years. These include the classical TDA^[^ ^15^ ^]^ to DLS,^[^ ^39^ ^]^ NMR,^[^ ^40^ ^]^ and native Mass spectrometry (MS) approaches such as field asymmetric ion mobility spectrometry (FAIMS)^[^ ^41^ ^]^ and trapped ion mobility spectrometry (TIMS).^[^ ^42^ ^]^ Despite their widespread application, each method presents notable limitations. TDA analysis can primarily study UV‐absorbing species, while often necessitating fluorescent labeling of solutes for their proper determination. DLS suffers from limited resolution in heterogeneous or polydisperse systems and is particularly sensitive to the presence of aggregates or impurities. NMR, while powerful, demands relatively high sample concentrations, specialized instrumentation, and significant technical expertise. FAIMS and TIMS are based on differential ion mobility and are reported to resolve multiple conformers of different proteins, but are limited in native‐like gas‐phase structures, and the diffusion coefficient is derived from models.

In light of these constraints, Calcagno et al.^[^ ^34^ ^]^ introduced a novel data analysis approach that deviates significantly from the original D‐SPR method based on the simplified Taylor–Aris assumption of ideal solutes. In their study, Calcagno and colleagues employed the Gillespie algorithm^[^ ^43^ ^]^ in conjunction with the discrete Fréchet distance (*d*_F_) to analyze the D‐SPR signal directly, thereby preserving the complete information embedded in the sensorgram—even for highly asymmetric signals generated by bovine serum albumin solutions in various chemical environments.

Their methodology hinges on generating a sufficiently large dataset of simulated curves with different sampling depths. Under these conditions, the determination of the *D* value is reformulated as a minimization problem, where the discrete Fréchet distance quantifies the difference between a simulated SPR curve, produced via stochastic simulations, and the average of multiple experimental D‐SPR sensorgrams.

As a result, this approach not only simplifies the extraction of *D* values but also reveals that different chemical environments lead to distinct unfolding processes and products. Specifically, by monitoring the temporal variation of *D* values, the study effectively contrasts SDS‐induced unfolding with pH‐induced unfolding pathways.

## 6 Summary and Outlook

In the previous paragraphs, we have described various applications of D‐SPR in its two variations for small and large diffusing entities, starting from the fundamental theory of Taylor‐Aris dispersion and extending it with the aid of modern stochastic and numerical simulations. These advances, combined with the ability of SPR instruments to create ideal experimental conditions, have significantly enhanced the method's applicability.

We demonstrated that carefully designed D‐SPR experiments enable the study and rationalization of the behavior of small linear molecules in water, even providing insights into their solute‐solvent intermolecular interactions. Furthermore, refinements to the method have facilitated the investigation of metal‐induced protein aggregation and protein folding, opening new avenues for studying folding‐unfolding transitions. This last statement gains considerable interest considering the impact that a thorough understanding of the very first phases of amyloidogenic proteins/peptides (e.g., seeding, oligomerization, and aggregation) would have in neurodegenerative and translational medicine. In fact, whilst amyloid deposition is a recognized and often pathognomonic signature of neurodegenerative processes of the brain (e.g., Alzheimer's and Parkinson's diseases) and of the optic‐nerve/retinal ganglion cells layer (e.g., glaucoma), the biophysical and chemical drivers are only partially understood. This is mostly due to the intrinsic technical and methodological limitations of monitoring aggregation processes in the liquid phase.

Nonetheless, targeting aggregation is still a therapeutic target for neurodegeneration, as emphasized by the growing development and testing of monoclonal antibodies (mAb) targeting soluble and insoluble amyloid aggregates.^[^ ^44^ ^]^

In this sense, the range of SPR‐based approaches for addressing problems in liquid phase diffusion remains vast and has not yet been fully explored. Future directions include the direct study of aggregation across different timescales in biological assemblies. A natural extension of both D‐SPR theory and experimental protocols would involve studying mixtures of multiple components—an area of critical importance in both biological and inorganic chemistry. Such an expansion could pave the way for a novel experimental framework to explore diffusion in highly nonideal conditions. Given an appropriate sensor for the species involved, D‐SPR experiments impose no fundamental concentration limits, provided a robust and theoretically sound data analysis protocol is available.

A particularly promising application in this context would be the study of amyloidogenic fibrils and their aggregation kinetics and equilibria in the presence and absence of metal ions.

## Conflict of Interest

The authors declare no conflict of interest.
